# Optimization on construction machinery considering sequence-dependent setup times and personnel fatigue based on the improved gray wolf and whale algorithm

**DOI:** 10.1371/journal.pone.0320753

**Published:** 2025-05-21

**Authors:** Dawei Wang, Bo Gao, Lei Zhang

**Affiliations:** 1 Key Laboratory of Metallurgical Equipment and Control Technology, Ministry of Education, Wuhan University of Science and Technology, Wuhan, China; 2 CCCC Second Highway Consultants Company Limited, Wuhan, China; 3 Wuhan Datong Engineering Construction Company Limited, Wuhan, China; 4 Precision Manufacturing Institute, Wuhan University of Science and Technology, Wuhan, China; Torrens University Australia, AUSTRALIA

## Abstract

In this study, the optimization of construction machinery scheduling within roadbed construction projects is explored, taking into account both personnel fatigue and sequence-dependent setup times. A sophisticated optimization model has been developed to simulate the optimal operation of machinery, aiming to maximize equipment utilization efficiency while addressing the challenges posed by worker fatigue. An innovative algorithm, the improved hybrid gray wolf and whale algorithm fused with a penalty function for construction machinery optimization (IHWGWO), is introduced, incorporating a penalty function to handle constraints effectively. This algorithm reduces the number of iterations required for optimization and, subsequently, cuts down on energy consumption. Through rigorous analysis and comparison with existing algorithms, the proposed IHWGWO demonstrates a significant reduction in both iteration count and financial expenditure. Simulation outcomes confirm the accuracy and practicality of the model and algorithm, establishing a promising new approach for scheduling in construction engineering.

## 1 Introduction

Highway construction projects often face tight time constraints, requiring efficient project management to minimize construction duration [1, 2]. Effective project management is essential for improving overall construction quality and meeting project timelines [3, 4]. According to statistics, the delay caused by unreasonable scheduling of construction machinery accounts for a large part of the total construction period in a highway construction project [5]. In addition, project budgets play a key role in project completion. Cost control measures must be put in place to ensure financial feasibility. The balancing of competing demands between time and cost is considered critical for the optimization of project effectiveness during construction. It is acknowledged that achieving time efficiency may necessitate the allocation of additional resources, equipment, skilled labor, and materials, consequently resulting in escalated construction costs [6]. Financial challenges and inadequate communication between management and general staff during the construction process contribute to a decrease in construction efficiency. Effective allocation of resources and scheduling, while adhering to established working procedures, can enhance construction efficiency and reduce costs throughout the construction process.

Roadbed engineering, a crucial component of highway engineering construction, involves extensive work and long timelines. The management of time and cost is crucial for the success of the project, requiring effective cost control, quality supervision, and schedule management. Traditional construction scheduling methods often depend on experienced personnel creating plans based on rough estimates, which can result in inefficiencies in machinery use and energy consumption, affecting construction progress, project costs, and overall quality [7]. In roadbed construction, the organization of processes and equipment scheduling is crucial. By strategically arranging processes and scheduling equipment, the subgrade construction task can be divided into steps like soil excavation, leveling, fine grading, compaction, and so on. Each step requires specific equipment with unique models and parameters. Geogrid technology is often used in highway construction for soil distribution to strengthen the soil, prevent displacement and deformation, and enhance overall stability. Excavators play a key role in soil distribution by quickly excavating earthworks for subsequent construction, thanks to their strong loading capacity for direct transport to designated areas. Bulldozers then evenly spread excavated earthworks in predetermined locations during the flattening process.

In order to achieve a high level of flatness for the ground, it is necessary to first use a bulldozer to roughen the surface and lay the foundation. This is followed by fine leveling and compaction to further improve flatness [8, 9]. The next step involves using a roller to accurately level the ground, ensuring a smooth finish. Finally, the ground is compacted using the weight and vibration function of the roller to meet design requirements for density and strength. It is important to establish the appropriate model of construction machinery for cost-effective and energy-efficient scheduling during the construction process.

Inefficient sequencing, stemming from order-dependent setup times, results in prolonged idle times and increased fuel consumption and emissions. In light of heightened environmental consciousness, the construction sector faces mounting pressure to implement sustainable practices aimed at reducing its ecological footprint. The optimization of construction machinery scheduling emerges as a critical strategy to address this challenge. This research advances the development of environmentally sustainable buildings by integrating sequence-dependent preparation times and personnel fatigue into a multi-objective scheduling model that incorporates environmental factors. This approach seeks to minimize resource consumption and mitigate pollution.

Extensive research has been conducted on construction machinery planning by scholars. R. Kuenzel et al. [10] explored the quantity of mechanical units in various road construction project categories, while Z. Zheng et al. [11] utilized data-driven unmanned construction sites to enhance overall construction efficiency. L. Xie et al. [12] applied case-based reasoning to address schedule delays in prefabricated building projects, and N. Essam et al. [13] investigated a multi-objective optimization approach for construction scheduling based on BIM. Researchers have studied diverse machinery types during highway construction, focusing on their utilization, quantity, configuration, and management. Mathematical modeling, simulation, and optimization algorithms have been employed to enhance mechanical planning efficiency. Environmental concerns such as energy conservation, emission reduction, and noise pollution mitigation have also been addressed in some studies. For example, X. Huang et al. [14] proposed an optimization design method for collaborative systems to reduce carbon emissions in projects by collaboratively optimizing the electrification layout of construction machinery. Although construction machinery scheduling methods have reported considerable success in those works, more attention is deserved to be paid on the comprehensive scheduling plan for construction machinery to maximize efficiency and minimize pollution levels. With the rapid development of technology, there is a growing integration of new intelligent, digital, and green technologies into construction machinery planning strategies. N. Wang et al. [15] proposed the development of a cooperative path for agricultural machinery and task assignments aimed at achieving overall green construction and pollution reduction objectives. G. Peng et al. [16] studied path prediction and design of bulldozers in construction machinery. It is shown that the critical need for further investigation and implementation of innovative approaches to address environmental concerns and improve efficiency in construction machinery operations.

Mechanical scheduling issues in construction have been studied, such as the optimization of scheduling plans for construction machinery in building assembly by L. Yang et al. [17]. However, there is a lack of research on optimizing equipment process scheduling in highway construction, particularly regarding the consideration of driver fatigue in the scheduling process. The objective of this paper is to integrate a model with an algorithm and optimize the model based on algorithmic characteristics. This process entails adjusting the algorithm input by establishing a connection between the model and the algorithm to better align it with the logic of the algorithm.

In order to enhance the optimization of engineering scheduling and establish a stronger theoretical foundation. Various methods have been employed to deal with the engineering scheduling problems. These methods, including the greedy algorithm, genetic algorithm, particle swarm algorithm, gray wolf algorithm, and simulated annealing algorithm, have been explored. The greedy algorithm constructs scheduling solutions incrementally by selecting locally optimal decisions at each step, providing simplicity and efficiency, although it may not always achieve the global optimal solution [18]. The genetic algorithm mimics natural selection and genetic mechanisms to explore and schedule solution spaces through crossover and mutation operations, showcasing robust global search capabilities and self-adaptability despite its high computational complexity. The simulated annealing algorithm imitates the metal annealing process to gradually converge towards the global optimal solution from a random starting point, demonstrating global search and escape capabilities from local extreme values, even though it may become trapped in local optima [19]. To overcome the limitations of individual algorithms, the proposal of hybrid algorithms, such as the gray wolf and particle swarm hybrid algorithm, has been observed within the academic discourse, as well as the genetic and particle swarm hybrid algorithm, to address these challenges.

Y. Chen et al. [20] proposed the use of the improved spider monkey optimization algorithm for multi-objective planning and scheduling on the PCB assembly line. X. Li et al. [21] optimized monthly interval scheduling for uncertain comprehensive process planning using a hybrid particle swarm optimization and genetic algorithm. P. Pabitha et al. [22] proposed a solution to cloud computing EI task scheduling uncertainty by utilizing chameleon and a search optimization algorithm. S. Wei et al. [23] and others employed an improved memetic algorithm for multi-objective resource-constrained flexible job shop inverse scheduling problem. Despite these advancements, there has been limited research on optimizing the layout scheduling algorithm for roadbed construction. Therefore, this paper suggests an improved gray wolf and whale hybrid algorithm to enhance the overall scheduling of roadbed construction machinery, aiming to address the current research gaps.

In future advancements, there is potential for further expansion and enhancement of optimization research in roadbed engineering as technology progresses and project management methods evolve [24]. By considering additional factors such as construction material selection and environmental protection, efforts can be made to strive for more sustainable and efficient roadbed construction. Additionally, by integrating emerging technologies such as artificial intelligence and big data, we can provide intelligent and precise project management solutions that lead to enhanced construction efficiency and quality [25].

The problem do not fully considered in the optimization algorithms and model discussed of the previous literature. To address this gap, this study introduces an improved hybrid gray wolf optimization algorithm fused with a penalty function for construction machinery optimization and whale algorithm. This algorithm strategically organizes equipment and machinery to minimize energy consumption and costs, drawing inspiration from humpback whale hunting behavior. By incorporating random numbers in two stages to enhance exploration capabilities, the algorithm achieves high performance, albeit potentially leading to longer processing times for complex problems. To improve computational efficiency, the algorithm leverages the internal mechanism of the gray wolf algorithm, capitalizing on hunting behavior and pack hierarchy. By proposing an optimal process configuration scheme upon completing the engineering task, the study aims to reduce energy consumption and costs while maintaining project quality and schedule. This algorithm and model can be utilized in various projects and can help streamline the scheduling process for many devices. This can result in reduced workload for initial preparations and cost savings for the project.

This paper presents a structured analysis of a novel optimization model developed for roadbed construction. The subsequent discussion is organized into four principal sections: First, Sect 1 furnishes a comprehensive exposition of the proposed optimization model, detailing its parameters and highlighting its novel features. Second, Sect 2 examines the optimization algorithms employed, providing a rigorous analysis of both the foundational algorithms and their subsequent refinements. Third, Sect 3 presents a comparative study of extant cases, with a critical evaluation of their corresponding results. Finally, Sect 4 concludes with a succinct summarization of the key research findings and the conclusions drawn from the investigation.

## 2 Optimization problem

### 2.1 Optization model

**Fig 1:**
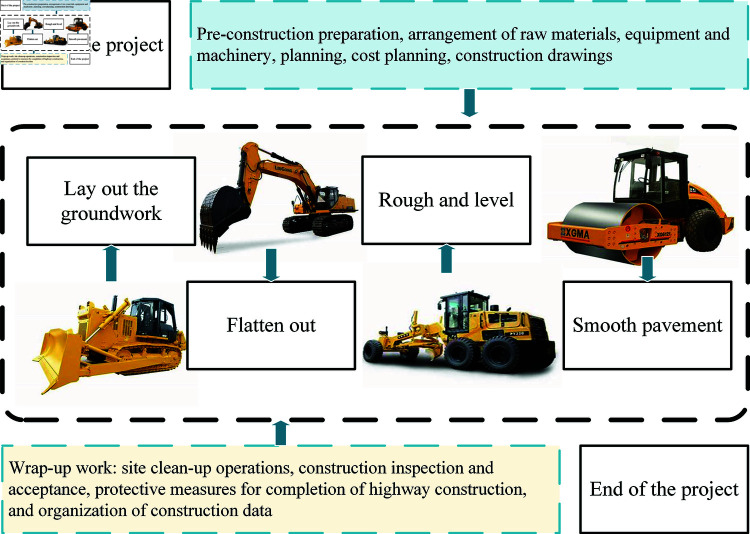
Overall process planning.

Following this structural overview, the focus shifts to the core of the optimization framework. The scheduling of roadbed construction can be likened to a parallel machine problem, similar to manufacturing workpieces in a workshop. The analysis of multiple machines should consider factors such as the type and size of engineering tasks, workload required for different processes, and equipment capacity. The model must adhere to constraints related to operation sequence and parallel processes. Operation sequence constraints dictate the order of construction processes, ensuring that the fixed sequence is followed. Parallel process constraints involve assigning tasks to available equipment simultaneously to maximize construction efficiency. However, the same equipment cannot be used in two processes simultaneously, it must complete one task before moving to the next. Mechanical matching ensures that each process is paired with suitable machinery for optimal performance. These considerations form the basis for building relevant models, the model is showed in Fig 1.

The order of the entire process remains unchanged, separate from the equipment and corresponding coding for jobs. It is important to ensure a reasonable arrangement of the overall process. The main task is to select model parameters based on the arrangement of equipment. Each working procedure of construction machinery is considered as a unit in the scheduling.

$$M=\{M_{11},\cdots ,M_{1i};M_{21},\cdots ,M_{2i};M_{31},\cdots ,M_{3i};M_{41},\cdots ,M_{4i}\}$$ (1)

where *M*_1_ is the excavator, *M*_2_ is the bulldozer, *M*_3_ is a grader, *M*_4_ is a road roller, i is the i model of the device. Similarly, each step in each task is treated as a unit.

$$T=\{T_{11},\cdots ,T_{1i};T_{21},\cdots ,T_{2i};T_{31},\cdots ,T_{3i}\}$$ (2)

where *T*_*ij*_ represents the j step of the i task.

To enhance the overall algorithmic model, an additional speed component is incorporated externally to the original cost function, along with the upper and lower limit constraints of the algorithm. Constraints that are not easily integrated directly into the model are represented through penalty values, thereby clarifying the framework of the overall model. The penalty factor is employed to designate the position of any violated constraint as a penalty.

The purpose of this study is to improve efficiency, less working time, and reduce pollution, and the objective function content of the final problem includes minimizing cost, minimizing time, and minimizing power consumption. The optimal operation problem is formulated as follows.

$$f_{n}(𝐱,M)=f(𝐱)+M\sum_{n=1}^{l}|c_{n}(x)|$$ (3)

$$\min y(x)=sum((\begin{array}{c} f_{1}(x),f_{2}(x),\cdots ,f_{n}(x) \end{array})),x\in \Omega^{n},M>0$$ (4)

where *y*(*x*) is the parameter to be minimized, including: cost, duration, power consumption and other functions, $f_{n}(𝐱,M)$ is the cost values corresponding to different parameters, *M* is the penalty factor, which represents the importance of different penalty values, *c*_*n*_(*x*) is the penalty function corresponding to the penalty value, depending on the following operational versus mechanical constraints, *n* is the number of costs as a measure. In order to ensure the integrity of the model and realize the effectiveness of the overall model, it is necessary to set the corresponding constraints.

(1) Operational constraints: all processes must have relevant equipment to do the work.

$$\sum_{x\in X}W_{xi}\leq N_{i},\forall i\in Y$$ (5)

where ${\text{W}}_{\text{xi}}$ is 1 if it is executed by machine x, and 0 otherwise, ${\text{N}}_{\text{i}}$ is the maximum number of equipment limitations for the i-th equipment. The first formula means that each process is known to have a corresponding equipment to work on. The second metric intends that the corresponding device for each process arrival is not greater than the maximum number of device limitations.

(2) Mechanical constraints: machinery can only perform one process at a time.

$$W_{xi}=\sum_{i\in {\hat{I}}_{0},i\neq j}Z_{ijx},\forall \nu \in X,j\in E_{\nu }$$ (6)

$$W_{xi}\geq \sum_{j\in \hat{I},j\neq i}Z_{ijx},\forall \nu \in X,i\in E_{\nu }$$ (7)

$$x_{x0}=1,\forall x\in X$$ (8)

$$\sum_{j\in \hat{I}}y_{0jx}\leq 1,\forall x\in X$$ (9)

where ${\text{Z}}_{\text{ijx}}$ is the machine x to execute job i and job j in this way is 1, otherwise it is 0.

where ${\text{M}}_{\text{i}}$ is the operating capacity of corresponding machinery per unit time $\left({\text{m}}^{\text{3}}/\text{h}\right)$, i respectively excavator, bulldozer, grader, roller, q is the excavator bucket capacity (${\text{m}}^{3}$), ${\text{K}}_{\text{p}}$ is the soil loosening coefficient. According to the soil level and excavator bucket capacity, take the value of 1, ${\text{K}}_{\text{H}}$ is the bucket filling coefficient. It is decided according to the soil level and bucket working mode, and takes the value of 0.75, n is the number of bucket working times per unit time of excavator, and takes the value of 100-300, E is the working efficiency, which is affected by many factors, p is the capacity of bucket of bulldozer (${\text{m}}^{3}$), t is the cycle time of tipper truck, it is the speed of the corresponding machinery per hour,${\text{V}}_{\text{i}}$ is the speed of the corresponding machinery per hour, g is the length of scraper of motor grader, ${\text{N}}_{\text{i}}$ is the number of corresponding machinery and equipment, l is the drum of road roller length.

The prismatic volume method used in the construction is used to calculate the amount of earthwork, and the random factors and soil types are considered to integrate the construction site. The cross section is regarded as composed of basic geometric figures (such as rectangles, triangles, trapezoids), and the dimensions of each part are measured respectively. The overall area and average height can be calculated. According to China’s highway engineering quality inspection and evaluation standards, the allowable deviation of subgrade width is ±5cm and the allowable deviation of elevation is ±3cm in general. The overall formula is as follows:

$$V=\frac{L}{6}\left(A_{1}+A_{2}+4A_{centre}\right)$$ (10)

where V is the earthwork volume between two cross sections, ${\text{A}}_{1}$ is the area of the first cross section, ${\text{A}}_{2}$ is the area of the second cross section, ${\text{A}}_{centre}$ is the cross section area at the midpoint of the two cross sections, and L is the distance between the two cross sections.

The total power of the equipment during construction is an important part of the overall model, which can reflect the energy consumption of the whole project, the total power is calculated by the number of each type of machinery to multiply the corresponding power of the equipment.

$$P=\sum_{1}^{i}n_{i}P_{i}H$$ (11)

where P is the total power consumed by the project construction machinery, ${\text{n}}_{\text{i}}$ is the number of type i devices, ${\text{P}}_{i}$ is the power of the i device, H is the working hours.

The time cost of highway subgrade construction encompasses expenses related to the scheduling and execution of construction activities [26]. This includes additional indirect costs resulting from delays in construction progress, such as capital occupancy expenses, extended equipment rental fees, heightened construction site management charges, and potential contract penalties. Time costs also encompass increased labor expenses due to prolonged construction durations, potentially necessitating overtime or off-hours work for workers. Moreover, project delays can impact the commencement of subsequent projects, leading to an overall increase in the project’s time cost. Therefore, effective time management and cost control are essential to safeguard the economic viability of highway subgrade construction.

Given the significance of time management, this study further investigates the application of parallel scheduling for minimizing the total time cost. To facilitate this analysis, a simplified model is proposed, wherein three distinct tasks are considered. Each task is comprised of four sequential processes ((*j*) = 1, 2, 3, 4), with each process corresponding to a unique equipment. A total of 12 kinds of equipment model (k = 1, 2,... 12), that is, each process corresponds to 3 equipment. The task(i,j) said the process j of task i in work. Make the ratio (i, j, k) said the device k of process j in task i . Set up each device type of workload for M(j) per hour. A task completion time calculation formula is as follows.

$$t_{1}(j,k)=\frac{task(1,j)\times ratio(1,j,k)}{\left(ratio(1,j,1)+ratio(1,j,2)+ratio(1,j,3))\times M(k\right)}$$ (12)

$$t_{1}(1)=\max\left(t_{1}(1,1),...,t_{1}(j,k)\right)$$ (13)

Let ${\text{t}}_{1}(j,k)$ denote the maximum completion time of process j within task 1. Here, j represents the equipment model utilized in the final operation of the process, indicating that the process has been completed and that the subsequent process associated with this task may now commence.

$$t_{2}(j,k)=t_{1}(j,k)+\frac{task(2,j)\times ratio(2,j,k)}{M(k)}$$ (14)

$$t_{2}(1)=\max\left(t_{1}(1,1)+t_{2}(1,1),...,t_{1}(j,k)+t_{2}(j,k)\right)$$ (15)

Within this context, the equipment that fulfills the requirements of the preceding task is scheduled to arrive ahead of time for the subsequent task, which involves the same process. The variable ${\text{t}}_{2}(1,\text{j})$ denotes the working time for process 1 at this juncture. It is established by the maximum value derived from the cumulative working times of the same process in both task 1 and task 2, respectively.

### 2.2 The sequence-dependent setup times consideration

In scheduling theory, sequence-dependent setup times refer to situations where the start time of a subsequent task or operation is influenced by the completion status of a preceding task or operation. The setup time is specific to the task order, meaning different sequences may lead to varying setup times. For construction machinery in highway engineering projects, each type of machinery has a unique setup time that depends on the sequence. Here, the sequence refers to the order of machinery rather than construction operations. The setup time for each machinery piece is determined by the completion time of the preceding operation, rather than just the predefined operation sequence. This flexible scheduling approach allows machinery to be scheduled based on the completion time of the preceding operation, reducing wait times and improving construction efficiency for the entire project.

$$S(1,j)=t_{1}(j-1),\;j>1$$ (16)

$$E(1,j)=t_{1}(j-1)+\max(t_{1}(j,k)),\;j>1$$ (17)

$$S(i,j)=\min\left(t_{i-1}(j,1),...,t_{i-1}(j,k)\right),\;i>1,j>1$$ (18)

$$E(i,j)=\max\left(t_{i-1}(j,1)+t_{i}(j,1),...,t_{i-1}(j,k)+t_{i}(j,k)\right),i>1,j>1$$ (19)

where S(i,j) is the initiation time of the j-th process associated with the i-th task, while E(i,j) signifies the commencement time of the j-th process of the i-th task, which is determined through the co-scheduling of the operational periods of preceding tasks across various machines. The temporal configuration pertaining to the sequence takes into account the adaptable scheduling of diverse working hours and machines, allowing for tasks to be executed without a rigidly enforced sequential dependency. Furthermore, identical types of machines may be concurrently utilized across different tasks.

### 2.3 Personnel fatigue consideration

While the above optimization focuses on efficiency and resource management, it is equally crucial to recognize the human element in construction. Specifically, this research recognizes the significance of maintaining safe working conditions for employees. Prolonged working hours coupled with inadequate rest intervals contribute to worker fatigue, which in turn results in a higher incidence of accidents, errors, and diminished efficiency on-site. Consequently, the management of personnel fatigue is not merely an ethical concern, but also a pragmatic necessity.

Building upon the consideration of sequence-dependent setup times, it is also crucial to account for the role of human factors in construction operations. In the operation of machinery, the performance is reliant on the staff, however, the efficiency of workers can be hindered by their own fatigue. As workers become more fatigued, their physical capabilities decline, subsequently reducing their work efficiency [27]. It is unrealistic to expect maximum efficiency from workers consistently over extended periods. It is important to consider the correlation between work efficiency and working hours. With the rise of intelligent manufacturing, the collaboration between humans and machines is becoming more common. Excessive fatigue during construction activities can have adverse effects on workers’ well-being and may also result in significant damage to the project. Therefore, it is essential to schedule workers’ hours based on their fatigue levels [28]. A well-structured schedule not only improves worker safety but also aids in minimizing construction timelines and optimizing resource utilization. This paper applies Konz’s exponential fatigue model [29].

This study not only uses the original fatigue index model but also evaluates the work efficiency of the workers. Researchers have employed utilizing pre-existing truck driver data, categorized the drivers based on factors such as age, lifestyle, and work experience. The research synthesizes the driver data in relation to prevailing national and social conditions and integrates indicators of work efficiency and temporal changes into the original calculation model. For general personnel, the work-rest schedule proposed by Casey GJ et al. is employed [30]. Adequate rest periods are required once work duration reaches a specified threshold to ensure workplace safety.

$$F^{W}(t_{i})=F^{W}(t_{i-1})+(1-F^{W}(t_{i-1}))(1-e^{-\lambda^{W}(t_{i}-t_{i-1})})$$ (20)

$$F^{w}(t_{i})=F^{w}(t_{i-1})e^{-\mu^{w}(t_{i}-t_{i-1})}$$ (21)

where ${\text{F}}_{\text{w}}({\text{t}}_{\text{i}})$ is the fatigue accumulation degree of worker w at the i moment, which ranges from 0 to 1, ${\text{F}}_{\text{w}}({\text{t}}_{\text{i}})$ is the residual fatigue after the length is $\text{t}\;-\;{\text{t}}_{i}$, λ and μ refer to the fatigue accumulation and recovery parameters of workers, which are the speed of fatigue accumulation and recovery, λ and μ range from (0.01, 0.03). They are defined as random variables subject to uniform distribution, and the initial fatigue is 0. On this basis, the workload on this model is converted according to the research of Y. S. Liu et al. [31].

$$r_{i,j,m}=1+\delta_{j}^{w}(\ln(1+F^{w}))\;$$ (22)

where ${\text{r}}_{i}$,j,m is the ratio between the actual work ability and the theoretical work ability, which can be multiplied by the corresponding theoretical work amount, that is, the actual work amount can be obtained after the final calculation.

**Fig 2:**
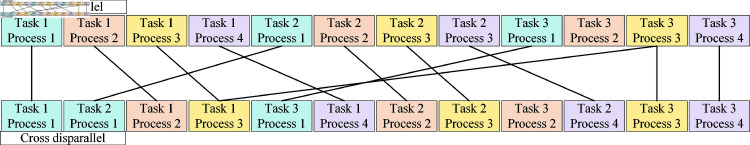
The interleaved processes.

As show in Fig 2, the first line represents the original process flow, and the second line represents the process flow after the process sequence change in order to achieve more efficient engineering construction.

**Table 1:** Vehicle-related parameters.

Machine name	Formula	Corresponding process
excavator	$M_{1}=\frac{nqK_{H}E}{K_{P}}$	lay out the groundwork
bulldozers	$M_{2}=\frac{npK_{H}E}{K_{P}}$	flatten out
land grader	$M_{3}=gv_{3}N_{3}$	rough and level
road roller	$M_{4}=lv_{4}N_{4}$	smooth pavement

## 3 Optimization algorithm

An improved hybrid gray wolf optimization algorithm fused with a penalty function for construction machinery optimization and whale algorithm for the optimization of project scheduling is introduced in the study, with the aim of enhancing the efficiency of the redevelopment phase of the whale algorithm. Search technology and random search technology are utilized by the whale algorithm itself to find prey by changing the position of each whale.

### 3.1 Primal algorithm

#### 3.1.1 WOA

The Whale Optimization Algorithm (WOA) is a metaheuristic optimization algorithm that emulates the hunting behavior of humpback whales. The primary distinctions of WOA compared to other swarm intelligence algorithms are the use of either random or best search agents to model prey searching behavior, and the implementation of a spiral mechanism to simulate the humpback whale’s bubble-net attack strategy [32]. The overall steps of the algorithm are as follows.

Step 1: Surround the prey:

$$\vec{X}(i+1)={\vec{X}}^{*}(i)-(2\cdot \vec{a}\cdot \vec{r}+\vec{a})\cdot \left|2\cdot \vec{r}\cdot {\vec{X}}^{*}(i)-\vec{X}(i)\right|$$ (23)

where X(i) is an iterative, is to find the best place i from the initial 2-0 until it reaches 0 at the end of the iterations, r is a random value of values range from 0 to 1, X(i+1) for the current position of whales. The following formula is used to calculate the distance between the best position and the current position and to create the spiral method.

$$\vec{X}(i+1)=e^{bk}\cdot \cos(2\pi k)\cdot \left|\vec{X^{*}}(i)-\vec{X}(i)\right|+\vec{X^{*}}(i)\;$$ (24)

where b is the constant that identifies the spiral shape, and k represents the random number in the range [-1,1], p is any number between 0 and 1. Combining the two structures above, each institution has a 50% chance of being selected:

$$\vec{X}(i+1)=\left\{ \begin{array}{cc} \vec{X^{*}}-2\cdot \vec{a}\cdot \vec{r}\cdot \left|\vec{X^{*}}(i)-\vec{X}(i)\right| & \text{if}\;p<0.5\\ e^{bk}\cdot \cos\left(2\pi k\right)\cdot \left|\vec{X^{*}}(i)-\vec{X}(i)\right|+\vec{X^{*}}(i) & \text{if}\;p\geq 0.5 \end{array}\right.$$ (25)

Step 2: Hunt for prey:

$$\vec{X}(i+1)=\vec{X_{rand}}-(2\cdot \vec{a}\cdot \vec{r}+\vec{a})\cdot \left|2\cdot \vec{r}\cdot \vec{X_{rand}}-\vec{X}\right|$$ (26)

${\text{X}}_{\text{rand}}$ iterates over time by evaluating the fitness of each agent based on the location of randomly selected whales within the range from which the whales surround their prey.

#### 3.1.2 GWO

Gray wolf algorithm has a social level according to the social level, and high to low were alpha, beta and delta, using the following formula to update the position of the prey [33].


$$\vec{D_{\alpha }}=\left|\vec{C_{1}}\cdot \vec{X_{\alpha }}-\vec{X}\right|$$

$$\vec{D_{\beta }}=\left|\vec{C_{2}}\cdot \vec{X_{\beta }}-\vec{X}\right|$$ (27)

$$\vec{D_{\delta }}=\left|\vec{C_{3}}\cdot \vec{X_{\delta }}-\vec{X}\right|$$

$${\vec{X}}_{1}=\vec{X_{\alpha }}-\vec{A_{1}}\cdot \vec{D_{\alpha }}$$

$${\vec{X}}_{2}=\vec{X_{\beta }}-\vec{A_{2}}\cdot \vec{D_{\beta }}$$ (28)

$${\vec{X}}_{3}=\vec{X_{\delta }}-\vec{A_{3}}\cdot \vec{D_{\delta }}$$

### 3.2 Improved algorithm (IHWGWO)

In order to improve the search efficiency of the whale algorithm, the search logic of the gray wolf algorithm is introduced.

$$\vec{X}(i+1)=\frac{\vec{X_{1}}+\vec{X_{2}}+\vec{X_{3}}}{3}$$ (29)

**Fig 3:**
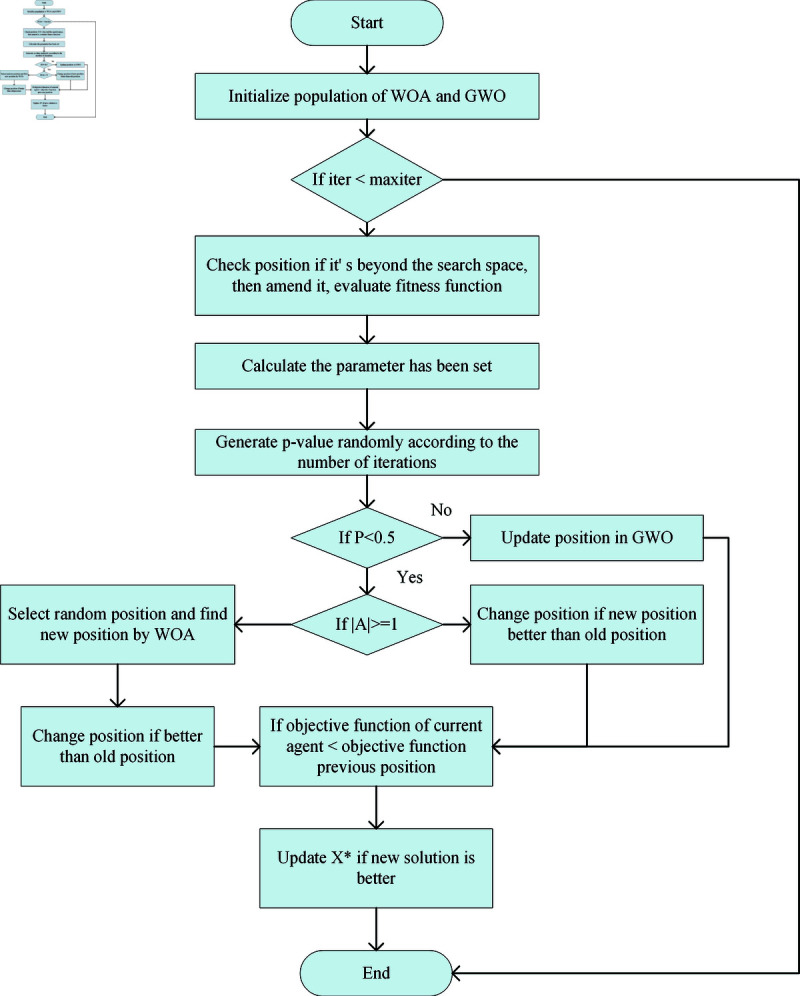
The process of IHWGWO.

**Fig 4:**
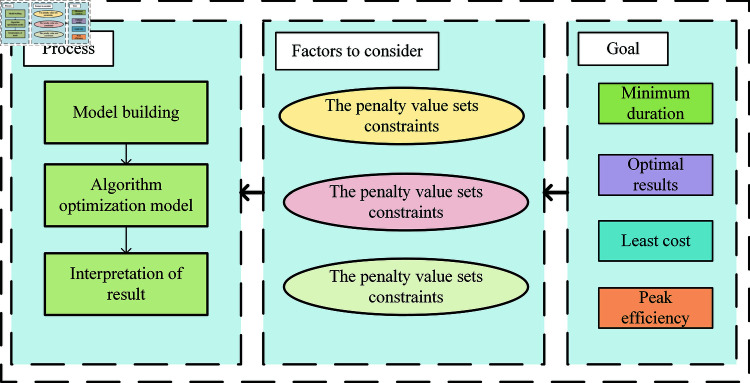
Model intrinsic parameter flow.

The updated position is re-evaluated in the fitness function, and the performance of the overall algorithm can be improved by adding the gray wolf algorithm[34]. Finally, the basic model flow of the mixed gray wolf whale algorithm can be obtained through the above formula. However, the above basic algorithm framework still has shortcomings. In order to make the algorithm and the model have better adaptability, In this paper, probabilistic cluster classification is added to the existing research to make the original p-value change with the number of iterations.

$$\text{p}=\left\{ \begin{array}{cc} p_{1} & iter<0.5*\text{maxIter}\\ p_{2} & iter\geq 0.5*\text{maxIter} \end{array}\right.$$ (30)

where *p*_1_ is a random number from 0.5 to 1, *p*_2_ is a random number from 0.1 to 0.4, and the p-value is limited by the number of iterations, so as to balance the search ability of the algorithm. In order to augment search efficiency, the enhancement of the improved hybrid gray wolf and whale algorithm (IHWGWO) is its integration of search agent methodologies from two distinct algorithms. This approach capitalizes on the strengths inherent in each algorithm. Specifically, the search agent capabilities of whale algorithm are employed during the initial iterations. Later, the multi-population grey wolf algorithm takes over for hybrid search. This shift occurs after satisfactory iteration results. In contrast to conventional algorithms, the IHWGWO employs a multi-stage search agent approach. Additionally, the adaptive mechanism of this algorithm tailors its strategies based on the number of iterations to enhance the search process. This integration of strategy significantly improves the overall efficiency and advancement of the algorithm. The overall process of the algorithm is as follows in Fig 3. And the overall hybrid algorithm pseudocode is embodied in Table 2. After combining the algorithm, the overall process and combination of this study are summarized as shown in Fig 4.

**Table 2:** Pseudocode of IHWGWO.

Initialize IHWGWO population *X_i_* where(*i=1,2,3,...,n*)
Update *a,A,C,L*
Evaluate the fitness function for each search agent
X* = the best search agent
**While** *iter < Maxiter*
**for each solution**
**If** iter< 0.5*Maxiter
**If** *|A|< 1*
Calculate new location of the present search agent by Eq (1)
**If** *y*(*x*)_*current*_ *<*y*(*x*)_*pervious*_*
Position = new_Position
**End**
**Else**
Change the location of the present search agent by Eq (2)
**If** *y*(*x*)_*current*_*<y*(*x*)_*pervious*_
Position = new_Position
**End**
**End**
**Else**
**If** *((A1 > -1 ||A1 < 1)&&(A2 > -1||A2 < 1)&&(A2 > -1||A3 < 1))*
Location of current search agent updated by Eq (29)
**End**
**End**
Return search agents to inside the search space if it goes beyond the search space
Fitness value for each search agent is calculated
Update X^*^ if there is a better solution *iter* = *iter* + 1
**End**
Return X^*^

## 4 Case study

### 4.1 Data of case

It is known that the subgrade project of a certain section of highway in China has three parts, the whole section of the subgrade project is 20*km* long and the width of the highway is 17.5*m*. Each part of the project can be simplified into four processes, the corresponding workload of each process is shown in Table 3. Additionally, Table 4 presents the mechanical properties and parameters for various models of different equipment. For this project, as mentioned in the model above, each process has corresponding equipment. And each device has the corresponding model and parameters, the formula in Table 1 is used to calculate the parameters of the known equipment, and the workload of the corresponding mechanical model is obtained.

**Table 3:** Vehicle-related parameters.

	Workplace organization	Process workload (*m^3^*)
Task one	Step one	3000
	Step two	1500
	Step three	9800
	Step four	9700
Task two	Step one	4000
	Step two	2000
	Step three	14000
	Step four	15000
Task three	Step one	6000
	Step two	3000
	Step three	19000
	Step four	20000

**Table 4:** Device parameter list.

Machine name	Equipment coding	Capacity (*m^3^/h*)	Cost (*yuan/h*)	Power (*kw*)
excavator	WYL100	180	918.52	130
	WLYL20	189	563.18	85
	CL815	270	1060.18	147
bulldozer	TL180A	400	735.97	120
	D80A-6	480	735.97	120
	D155A-1A	700	1478.75	235
grader	PY160B	66	497.36	118
	PY160C	62	294.16	110
	GD511A-1	67	897.33	101
road roller	SPR300C-10H	708	502.87	140
	SSR220-AC-10H	1278	811.92	155
	STR130C-10C	854	527.43	125

### 4.2 Computational results of the case

**Fig 5:**
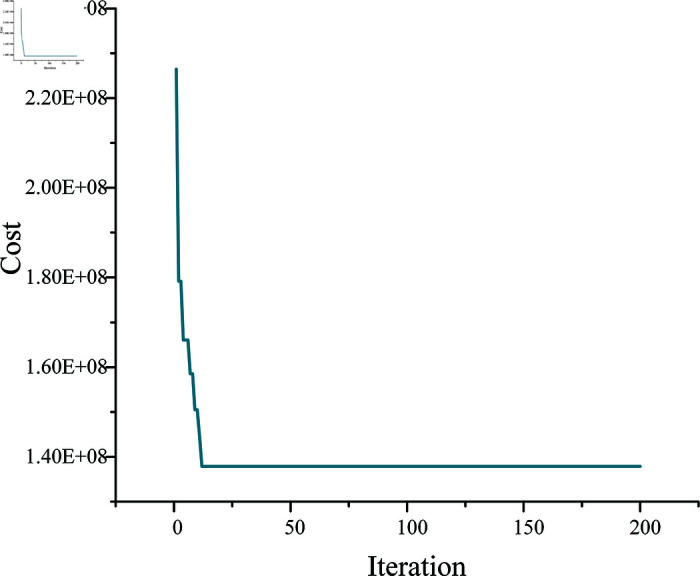
Final optimization results.

Having established the parameters of the project. The IHWGWO algorithm and constructive heuristic method were utilized in this study. The computational results are presented in Fig 5. To investigate the CPU time of the proposed method, the time performance of the proposed IHWGWO was calculated. Experiments were performed using Python 3.9 on an AMD Ryzen 7 7840H w CPU with 16 GB of RAM. Python was employed for calculations, with 200 iterations taking five minutes. The results consistently decreased with increasing iterations, eventually converging to a minimum value, representing the optimal solution. This optimal result was achieved by considering all constraints of the model, including the fatigue of construction personnel and the independent time dependence of the process.

Furthermore, this paper explores the overall scheduling of highway subgrade construction. The establishment of the model recognizes the importance of both parallel and time-series characteristics of mechanical operations, which significantly influence the solution. Additionally, the incorporation of sequence-dependent setup times provides a more realistic framework for evaluating how equipment arrangement impacts the total working time. By adopting a parallel scheduling approach with consideration of sequence-independent setup times, the study demonstrates a flexible methodology for arranging construction machinery, ultimately achieving a substantial reduction in the overall project duration. Moreover, in addition to examining the constraints associated with process time, this study also incorporates an analysis of worker fatigue as an essential factor influencing the model.

**Fig 6:**
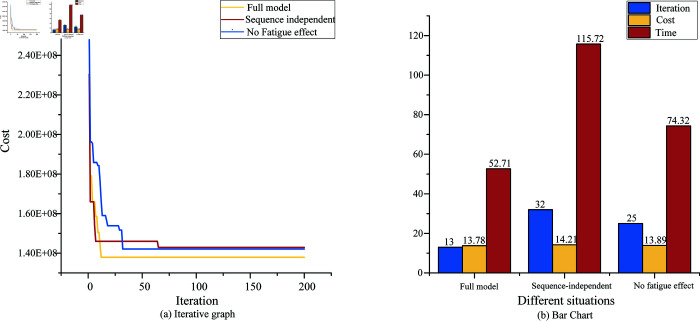
The computational results of the case.

Fig 6 shows the comparison of the results of the model that takes sequence-dependent setup times and worker fatigue into account and the model that does not take into account the two factors. The IHWGWO is utilized to optimize the model, with Fig 6 (a) presenting a comparison of algorithm iterations with and without considering sequence-dependent setup times, and Fig 6 (b) providing a comparison of their respective outcomes. The specific optimization time value is shown in Table 5. This methodology results in a significant reduction of almost 50% in overall working time when compared to scheduling without sequence-dependent setup times. The advantages of parallel scheduling for sequence-dependent setup times are highlighted.

**Table 5:** Working time of different processes (*h*).

	Sequence dependent			Sequence dependent		
	Task 1	Task 2	Task 3	Task 1	Task 2	Task 3
Step 1	13.1	18.8	31.8	16.3	25	40.2
Step 2	2.6	3.9	5.1	3.2	4.7	6.2
Step 3	14.7	21	28.6	19.6	29.5	40.2
Step 4	12.1	17.8	21.5	14.7	24.3	23.2

**Fig 7:**
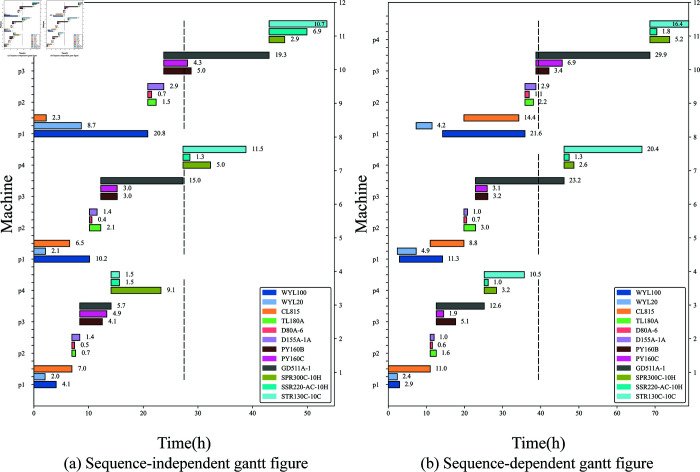
The comparison in gantt result.

In Fig 7, Fig 7 (a) displays the scheduling gantt chart for sequence-independent setup times, while Fig 7 (b) shows the scheduling gantt chart for sequence-dependent setup times. Due to the requirements of sequence-independent setup times, the overall time is too long to be easily graphed, resulting in the three tasks being displayed on a horizontal line. However, the total computation time is the sum of the final completion times of the three tasks, which amounts to 115.72 hours in this study. Conversely, with sequence-dependent setup times, flexible scheduling of construction machinery leads to a significant reduction in overall working time. The overall working time at the end of scheduling is 85.04 hours. In the previous Fig 6 histogram results, without considering fatigue and the sequence-dependent setup times, the final time is 52.71 hours. The final results indicate that despite the consideration of sequence-independent setup times, fatigue has a lesser impact on overall scheduling results compared to time series consideration, showcasing the superiority of sequence-independent setup times for engineering optimization.

### 4.3 Comparison with various algorithms

**Fig 8:**
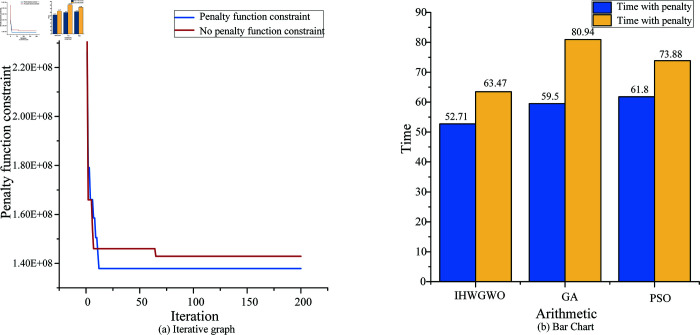
Optimize comparison of whether cto set penalty values.

In order to guarantee the rationality of the whole model, the model of adding the corresponding punishment within the value used to limit the model parameters is not beyond the scope of the right, to get more reasonable results, for algorithm can better realize the final convergence, the optimal result is shown in Fig 8.

The penalty value is set to enhance the constraints and improve the gray wolf whale algorithm. Fig 8 illustrates the impact of device penalty values on this model, with Fig 8 (a) being an iterative image map and Fig 8 (b) showing a comparison of algorithm results. It also allows for a comparison of penalty values among different algorithms, such as genetic algorithm, particle swarm algorithm, and IHWGWO, to observe changes in the total time arrangement. Table 6 compares the algorithm results in different cases. Setting a penalty value is beneficial for reducing the final time arrangement and energy consumption.

**Table 6:** A comparison of whether to add a penalty.

	Time with penalty (*h*)	Time without penalty (*h*)
IHWGWO	52.71	63.47
GA	59.5	80.94
PSO	51.75	73.88

Additionally, on the premise of satisfying the integrity of the model, the improved gray wolf whale algorithm is compared with other algorithms to compare whether it has advantages. This model not only has a fast iteration speed, but also can get a better result after the algorithm optimization, that is, the minimum cost and the highest efficiency.

**Fig 9:**
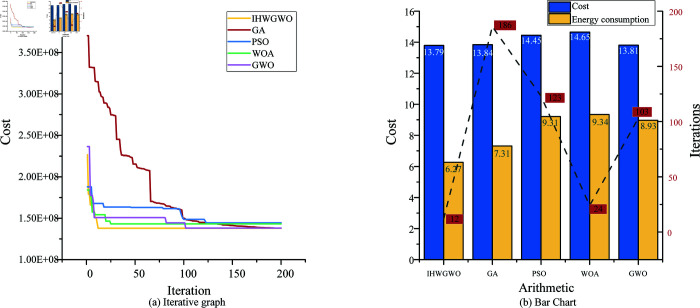
Multi-algorithm comparison figure.

In order to systematically establish the superiority of the proposed algorithm, a comparative analysis was performed involving four alternative algorithms. The final engineering construction cost and the number of algorithm iterations were analyzed. As shown in Fig 9 (a), the cost of the overall engineering project construction varied significantly. Additionally, Fig 9 (b) illustrates that the improved gray wolf and whale algorithm not only had the lowest cost and the minimum energy consumption but also required the fewest iterations. Finally, the optimization performance of different algorithms is shown in Table 7. The implementation of optimized scheduling led to a decrease of over 14.2% in projected fuel consumption, which was accompanied by a proportional decline in CO_2_ emissions. This finding underscores the potential for substantial environmental advantages associated with this optimized scheduling methodology.

**Table 7:** Comparison of different algorithms.

	Cost	Energy consumption	Iterations
IHWGWO	13.79	6.27	12
GA	13.84	7.31	186
PSO	14.45	9.21	123
WOA	14.65	9.34	24
GWO	13.81	8.93	103

## 5 Conclusions

In order to reduce the cost and energy consumption in the construction process of roadbed, a multi-objective scheduling optimization approach is presented with various constraints, such as parallel operation constraint, personnel fatigue constraint, and overall workload constraint. These constraints utilize penalty values to specify the intensity for the parallel operation arrangements and the personnel fatigue. With the characteristics of the optimization model, the IHWGWO algorithm is presented to achieve flexible machinery scheduling in the roadbed construction which allows for parallel operation rather than the sequence-independent setup times. The case study demonstrates that the proposed IHWGWO algorithm can achieve cost-effective and energy-efficient scheduling while maintaining construction completion rates. Due to this paper not only considers machinery parameters but also incorporates parallel operation and personnel fatigue with a more comprehensive model, the proposed IHWGWO algorithm can lessen the iterations and construction cost for the optimization problem compared with other algorithms. In addition, the IHWGWO can reduce the energy consumption of equipment and reduce environmental pollution more than other algorithms.

## Supporting information

S1 File Optimize process data and final results (XLSX)

## References

[1] Ibrahim AH, Shaker MA. Sustainability index for highway construction projects. Alex Eng J. 2019;58(4):1399–411. doi: 10.1016/j.aej.2019.11.011

[2] de Bortoli A, Baouch Y, Masdan M. BIM can help decarbonize the construction sector: Primary life cycle evidence from pavement management systems. J Clean Prod. 2023;391:136056. doi: 10.1016/j.jclepro.2023.136056

[3] Antoine ALC, Molenaar KR. Latent class analysis for highway design and construction project categorization. Proced Eng. 2016;145:1314–21. doi: 10.1016/j.proeng.2016.04.169

[4] Nohair L, Adraoui AE, Namir A. An improved hybrid metaheuristic for active job-shop scheduling problems. Proced Comput Sci. 2024;231:56–62. doi: 10.1016/j.procs.2023.12.164

[5] Tariq J, Shujaa Safdar Gardezi S. Study the delays and conflicts for construction projects and their mutual relationship: A review. Ain Shams Eng J. 2023;14(1):101815. doi: 10.1016/j.asej.2022.101815

[6] Liu Y, You K, Jiang Y, Wu Z, Liu Z, Peng G, et al. Multi-objective optimal scheduling of automated construction equipment using non-dominated sorting genetic algorithm (NSGA-III). Autom Construct. 2022;143:104587. doi: 10.1016/j.autcon.2022.104587

[7] Li J, Liao C, Xiong C, Chen C, Wang Z, Wu C, et al. Research on distresses detection, evaluation and maintenance decision-making for highway pavement in reconstruction and expansion project. Case Stud Construct Mater. 2023;19:e02451. doi: 10.1016/j.cscm.2023.e02451

[8] Wang X, Dong X, Li J, Zhang Z, Zhang J, Ma G. Developing an advanced ANN-based approach to estimate compaction characteristics of highway subgrade. Adv Eng Inform. 2023;56:102023. doi: 10.1016/j.aei.2023.102023

[9] Liu W, Huang X, Feng X, Xie Z. Compaction and bearing characteristics of untreated and treated lateritic soils with varying moisture content. Construct Build Mater. 2023;392:131893. doi: 10.1016/j.conbuildmat.2023.131893

[10] Kuenzel R, Teizer J, Mueller M, Blickle A. SmartSite: Intelligent and autonomous environments, machinery, and processes to realize smart road construction projects. Autom Construct. 2016;71:21–33. doi: 10.1016/j.autcon.2016.03.012

[11] Zheng Z, Wang F, Gong G, Yang H, Han D. Intelligent technologies for construction machinery using data-driven methods. Autom Construct. 2023;147:104711. doi: 10.1016/j.autcon.2022.104711

[12] Xie L, Wu S, Chen Y, Chang R, Chen X. A case-based reasoning approach for solving schedule delay problems in prefabricated construction projects. Autom Construct. 2023;154:105028. doi: 10.1016/j.autcon.2023.105028

[13] Essam N, Khodeir L, Fathy F. Approaches for BIM-based multi-objective optimization in construction scheduling. Ain Shams Eng J. 2023;14(6):102114. doi: 10.1016/j.asej.2023.102114

[14] Huang X, Huang Q, Cao H, Yan W, Cao L, Zhang Q. Optimal design for improving operation performance of electric construction machinery collaborative system: Method and application. Energy. 2023;263:125629. doi: 10.1016/j.energy.2022.125629

[15] Wang N, Yang X, Wang T, Xiao J, Zhang M, Wang H, et al. Collaborative path planning and task allocation for multiple agricultural machines. Comput Electron Agric. 2023;213:108218. doi: 10.1016/j.compag.2023.108218

[16] Peng G, Duan H, Tan Z, Zhou Y, Li J, Hu B, et al. Construction path tracking and pose estimation of unmanned bulldozer. Autom Construct. 2023;154:105015. doi: 10.1016/j.autcon.2023.105015

[17] Liu Y, Ma X, Jiang M, Huang W, Ren H. Location selection of agricultural Machinery sheds for improved scheduling and efficiency under sustainability goals. Ecol Indicat. 2023;155:110986. doi: 10.1016/j.ecolind.2023.110986

[18] Feng X, Zhao F, Jiang G, Tao T, Mei X. A tabu memory based iterated greedy algorithm for the distributed heterogeneous permutation flowshop scheduling problem with the total tardiness criterion. Expert Syst Appl. 2024;238:121790. doi: 10.1016/j.eswa.2023.121790

[19] Ozcan-Deniz G, Zhu Y. Multi-objective optimization of greenhouse gas emissions in highway construction projects. Sustain Cities Soc. 2017;28:162–71. doi: 10.1016/j.scs.2016.09.009

[20] Chen Y, Zhong J, Mumtaz J, Zhou S, Zhu L. An improved spider monkey optimization algorithm for multi-objective planning and scheduling problems of PCB assembly line. Expert Syst Appl. 2023;229:120600. doi: 10.1016/j.eswa.2023.120600

[21] Li X, Gao L, Wang W, Wang C, Wen L. Particle swarm optimization hybridized with genetic algorithm for uncertain integrated process planning and scheduling with interval processing time. Comput Ind Eng. 2019;135:1036–46. doi: 10.1016/j.cie.2019.04.028

[22] Pabitha P, Nivitha K, Gunavathi C, Panjavarnam B. A chameleon and remora search optimization algorithm for handling task scheduling uncertainty problem in cloud computing. Sustain Comput: Inform Syst. 2024;41:100944. doi: 10.1016/j.suscom.2023.100944

[23] Wei S, Tang H, Li X, Lei D, Wang XV. An improved memetic algorithm for multi-objective resource-constrained flexible job shop inverse scheduling problem: An application for machining workshop. J Manuf Syst. 2024;74:264–90. doi: 10.1016/j.jmsy.2024.03.005

[24] Zheng Z, Wang F, Gong G, Yang H, Han D. Intelligent technologies for construction machinery using data-driven methods. Autom Construct. 2023;147:104711. doi: 10.1016/j.autcon.2022.104711

[25] Liu X. Mathematical scheduling model of complex industrial process combining swarm intelligence algorithm and swarm dimension reduction technology. Results Eng. 2024;21:101796. doi: 10.1016/j.rineng.2024.101796

[26] Liu Y, Zhang Z, Zhu D, Bo L, Yang S, Yue Y, et al. Mine water cooperative optimal scheduling based on improved genetic algorithm. Heliyon. 2024;10(6):e27289. doi: 10.1016/j.heliyon.2024.e27289 38510030 PMC10950494

[27] Kang J, Payne SC, Sasangohar F, Mehta RK. Field-based longitudinal evaluation of multimodal worker fatigue assessments in offshore shiftwork. Appl Ergon. 2024;115:104164. doi: 10.1016/j.apergo.2023.104164 37925754

[28] Darwish MA. Optimal workday length considering worker fatigue and employer profit. Comput Ind Eng. 2023;179:109162. doi: 10.1016/j.cie.2023.109162

[29] Konz S. Work/rest: Part II – The scientific basis (knowledge base) for the guide. Int J Ind Ergon. 1998;22(1–2):73–99. doi: 10.1016/s0169-8141(97)00069-3

[30] Casey GJ, Miles-Johnson T, Stevens GJ. Heavy vehicle driver fatigue: Observing work and rest behaviours of truck drivers in Australia. Transport Res Part F: Traffic Psychol Behav. 2024;104:136–53. doi: 10.1016/j.trf.2024.05.016

[31] Liu Y, Shen W, Zhang C, Sun X. Agent-based simulation and optimization of hybrid flow shop considering multi-skilled workers and fatigue factors. Robot Comput-Integr Manuf. 2023;80:102478. doi: 10.1016/j.rcim.2022.102478

[32] Mirjalili S, Lewis A. The whale optimization algorithm. Adv Eng Softw. 2016;95:51–67. doi: 10.1016/j.advengsoft.2016.01.008

[33] Mirjalili S, Mirjalili SM, Lewis A. Grey wolf optimizer. Adv Eng Softw. 2014;69:46–61. doi: 10.1016/j.advengsoft.2013.12.007

[34] Mohammed H, Rashid T. A novel hybrid GWO with WOA for global numerical optimization and solving pressure vessel design. Neural Comput Appl. 2020;32(18):14701–18. doi: 10.1007/s00521-020-04823-9

